# Influence of App-Based Self-Management on the Quality of Life of Women With Endometriosis

**DOI:** 10.7759/cureus.67655

**Published:** 2024-08-24

**Authors:** Nadine Rohloff, Teresa Götz, Sarah S Kortekamp, Nicole R Heinze, Charlotte Weber, Sebastian D Schäfer

**Affiliations:** 1 Science, Endo Health GmbH, Chemnitz, DEU; 2 Gynecology and Obstetrics, Clemenshospital, Münster, DEU

**Keywords:** endometriosis, digital therapeutics, self-management counseling, quality of life (qol), health app, endometriosis and chronic pelvic pain

## Abstract

Introduction: Endometriosis can significantly impair the quality of life of those affected. Multimodal self-help measures are recommended but are often difficult to access. Smartphone apps have been shown to improve the quality of life for other conditions with chronic pain. The aim of this study was to examine the impact of the Endo-App (Endo Health GmbH, Chemnitz, Germany) on both disease-related quality of life and symptoms of endometriosis affecting it.

Methods: In the present randomized, controlled pilot study, the impact of utilizing the Endo-App on the quality of life among a sample of 122 women affected by endometriosis is assessed. To measure the changes over a 12-week period, the study incorporates the validated Endometriosis Health Profile (EHP-5 and EHP-30) questionnaire from Oxford University, among other assessment tools.

Results: The use of the Endo-App leads to significant changes in the following areas after 12 weeks compared to the control group: pain disability, pain self-efficacy, fatigue, depressive symptoms, and Endometriosis Health Profile scores. The EHP-5 score from T0 to T12 is reduced by -16.76 (p-value of the Mann-Whitney U test (p_U_) = 0.008), and the EHP-30 score by -15.48 (p_U_ = 0.004). The results remain significant in sensitivity analyses. The effect size of Cohen's d was in the medium range.

Conclusion: In summary, the Endo-App improves both physical and psychological symptoms and the patient's self-efficacy. The Endo-App contributes to improving endometriosis care in Germany and enables women suffering from endometriosis to significantly increase their quality of life.

## Introduction

Approximately one in 10 women of childbearing age in Germany, around 1.7 million individuals, suffer from endometriosis [1]. This chronic disease can significantly impact the quality of life of those affected. Common symptoms include severe menstrual pain and non-cyclic lower abdominal pain, along with other symptoms and functional complaints depending on the location of endometriosis lesions. It often takes several years until a diagnosis is made. In addition, the cause of the disease is still unclear and there are high recurrence rates with current therapies [1]. Prolonged symptoms can lead to complex neurological changes that contribute to the development of chronic pain [2].

In a consensus paper on the treatment of chronic pain syndromes, experts from the German Pain Society recommend that patients with chronic pain, regardless of its origin, be educated on self-help measures [3]. Exercise therapy, nutritional therapy, and stress-reducing concepts as well as the provision of information and the use of symptom diaries are recognized as universally effective options [4]. Despite alignment among expert recommendations, guidelines, and reviews [3], options for these interventions are limited in Germany.

Studies have demonstrated that smartphone apps can successfully integrate these therapy supplements into the daily lives of individuals with various chronic pain conditions, leading to positive effects on pain, symptoms, and overall management of the condition [5]. Digital self-management concepts are already being used to support the management of other chronic diseases [6].

To apply such a concept specifically for endometriosis, the Endo-App (Endo Health GmbH, Germany) was developed as a medical product that utilizes a multimodal, interdisciplinary approach to endometriosis therapy. The app incorporates information provision, psychosocial support, exercise therapy, nutritional therapy, stress reduction techniques, and coping strategies to complement standard therapy, aiming to contribute to a significant improvement in the quality of life of those affected [7].

The objective of this study was to evaluate the influence of the Endo-App on disease-related quality of life and symptoms of endometriosis affecting it, in comparison with a control group (waitlist design).

This article was previously presented as a poster at the 15th World Congress on Endometriosis, from May 3 to 6, 2023.

## Materials and methods

The appropriate ethical approval by the Ethics Committee of the State Chamber of Physicians of Saxony, Germany was received (EK-BR-113/21-1) and appropriate processes were followed. The study was completed in accordance with the Declaration of Helsinki as revised in 2013.

Study design

To investigate the impact of the Endo-App on disease-related quality of life and quality-of-life-affecting symptoms of endometriosis, a two-arm randomized controlled trial with a waitlist design was conducted. The participants were randomly assigned to either the intervention group or the control group.

Measuring instruments

To investigate the effect on health-related quality of life (HrQoL), the validated Endometriosis Health Profile (EHP) questionnaire was used [8,9].

To evaluate possible other effects, the participants were also asked to complete further questionnaires, including validated questionnaires like Fragebogen zur Erfassung der schmerzspezifischen Selbstwirksamkeit (FESS), Pain Disability Index (PDI), Fatigue Severity Scale (FSS), and Patient Health Questionnaire (PHQ-9), and questions about demographics and daily life effects (Table 1).

**Table 1:** Instruments used to measure the effect of the Endo-App.

Name of the instrument/scale	Content
Endometriosis Health Profile (EHP) - 30 items (EHP-30), EHP - 5 items (EHP-5)	Assessment of the influence of endometriosis on the life of those affected on a scale from "Never" to "Always." Scale ranges from 0 to 100. Validated questionnaires, German version [8]. A translation certificate from Oxford University Innovation is available for the EHP-5 and the EHP-30.
Endometriosis Health Profile: EHP Work, EHP Sex, EHP Doctor	Assessment of the influence of endometriosis on working life, sexual life, and the relationship to doctors of patients on a scale from "Never" to "Always."
Fragebogen zur Erfassung der schmerzspezifischen Selbstwirksamkeit (FESS)	Assessment of self-efficacy expectations in patients with chronic pain on a scale of 0-6. Developed from the Pain Self-Efficacy Questionnaire (PSEQ). Validated questionnaire, German version [10].
Pain Disability Index (PDI)	Assessment of the impairment in everyday life due to pain in seven areas of life on a scale of 0-10 ("None" to "Total impairment"). Validated questionnaire, German version [11].
Fatigue Severity Scale (FSS)	Assessment of chronic fatigue on a scale of 1-7 ("Strongly disagree" to "Strongly agree"). Validated questionnaire, German version [12,13].
Patient Health Questionnaire (PHQ-9)	Assessment of depression on a scale of 1-4 ("Not at all" to "Almost every day"). Validated questionnaire, German version [14].
Generalized Anxiety Disorder Scale (GAD-7)	Assessment of generalized anxiety disorder on a scale of 1-4 ("Never" to "Almost every day") [15].
Health Education Impact Questionnaire (HeiQ)	Assessment of health-related self-management skills after education or rehabilitation. Validated questionnaire, German version [16].
Incapacity to work	Assessment of periods of incapacity to work due to endometriosis.
Life satisfaction	Assessment of life satisfaction.
Symptoms of endometriosis	Assessment of the state of endometriosis and distress.
Long-term cycle	Assessment of the long-term cycle.
Distraction score	Assessment of the extent of distraction during the online survey.

Study population: Inclusion and exclusion criteria

The inclusion criteria for this study comprised a medical diagnosis of endometriosis (N80 - N80.9), legal capacity, age over 18 years, informed consent, female sex, residence in Germany, and sufficient knowledge of the German language.

Exclusion criteria included current pregnancy or undergoing medical treatment for infertility, surgery within the past two months, and plans for a new therapy or change of therapy (including surgery or hormonal changes) during the study period. All inclusion and exclusion criteria were assessed through self-reporting in the questionnaire.

Study procedure

After completing the baseline questionnaire, simple, unrestricted randomization was carried out directly in the online tool SoSci Survey.

After inclusion and randomization, the 12-week study period followed. The intervention consisted of 12 weeks of active use of the Endo-App as an add-on to the ongoing treatment of standard care (care-as-usual). The control group received care as usual exclusively.

The participants received the link to the follow-up surveys with the questionnaires after four, eight, and 12 weeks.

Statistical methods

Statistical analyses were conducted using SPSS (IBM SPSS Statistics for Windows, Version 22.0., IBM Corp., Armonk, NY). Exploratory evaluations of change scores in the questionnaires were performed. The data were analyzed pseudonymously.

Changes within the groups from baseline (T0) to four weeks (T4), eight weeks (T8), and 12 weeks (T12) were assessed using Wilcoxon tests. To compare change scores between the groups, the Mann-Whitney U test was utilized. Non-parametric tests were chosen due to the potential non-normal distribution of quality-of-life questionnaires like the EHP, ensuring robustness in the analysis [9].

For the primary analysis of all endpoints, a complete case analysis was performed, including all participants who completed the online survey at both T0 and T4/8/12. A sensitivity analysis with baseline-observation-carried-forward (BOCF) imputed datasets corresponding to an intention-to-treat (ITT) analysis was conducted. The evaluation included all participants who met the inclusion and exclusion criteria, completed the online survey at T0, and were randomized. Missing values were replaced with the imputation procedure BOCF. This conservative evaluation was performed for all sum values of the validated questionnaires (EHP-5, EHP-30, FESS, PDI, PHQ-9, FSS, and Generalized Anxiety Disorder Scale (GAD-7)).

## Results

Demographics

The study population consisted of 122 randomized women who met all inclusion and no exclusion criteria and answered the first questionnaire completely. A total of 85 persons did not meet the inclusion criteria of the study or met exclusion criteria, e.g., reported being male or having no residence in Germany. Of the remaining 152 women, 30 did not complete the first questionnaire.

Respondents were aged between 20 and 52 years, with a mean of 29.6 years. There were no significant differences between the intervention group and the control group in terms of age (p-value of the Mann-Whitney U test (p_U_) = 0.945), employment status (p-value of the chi-square test (p_Chi_) = 0.351), reported symptoms (each p-value of the Fisher's exact test (p_exF_) > 0.1), days with pain medication (p_U_ = 0.572), hysterectomy status (one in each group, p_exF_ = 1), cycle timing (p_U_ = 0.358), hormonal treatment status (each p_exF_ > 0.1), or their periods of incapacity to work (p_U _= 0.966). There were also no significant differences regarding the questionnaire results recorded at baseline (each p_U_ > 0.1).

The study recorded how the participants received their endometriosis diagnosis. Multiple answers were possible. The most common diagnostic method was by means of surgery, which was indicated by 92% in the intervention group and 88.9% in the control group. Every participant was diagnosed by a physician. There were no significant differences in the percentage of endometriosis diagnostic procedures between the intervention and control groups (p_exF_ > 0.7 in each case).

Overview of change scores and effect sizes

Table 2 shows an overview of the change scores of the recorded questionnaires.

**Table 2:** Overview of change scores of the questionnaires. ^a^ P(u) = P-value of the Mann-Whitney U test. ^b^ EHP-5 = Endometriosis Health Profile - 5 items. ^c^ EHP-30 = Endometriosis Health Profile - 30 items. ^d^ PDI = Pain Disability Index. ^e^ FESS = Fragebogen zur Erfassung der schmerzspezifischen Selbstwirksamkeit. ^f^ FSS = Fatigue Severity Scale. ^g^ PHQ-9 = Patient Health Questionnaire. ^h^ GAD-7 = Generalized Anxiety Disorder Scale.

Parameter	Endo-App		Control			P(u)^a^
	T12-T0		T12-T0			
	Number	Mean (standard deviation)	Number	Mean (standard deviation)	Mean (standard error of difference)	
EHP-5^b^	34	-16.76 (17.66)	64	-6.72 (15.67)	10.04 (3.48)	0.008
EHP-30^c^	34	-15.48 (15.19)	63	-6.32 (12.95)	9.15 (2.93)	0.004
PDI^d^	32	-8.09 (9.91)	57	-1.82 (11.00)	6.27 (2.35)	0.007
FESS^e^	33	5.73 (7.66)	62	1.95 (8.25)	3.78 (1.74)	0.029
FSS^f^	34	-3.88 (9.11)	61	1.69 (6.39)	5.57 (1.60)	0.003
PHQ-9^g^	34	-3.03 (3.74)	62	0.21 (3.75)	3.24 (0.80)	< 0.001
GAD-7^h^	34	-2.26 (4.05)	61	-0.74 (3.49)	1.53 (0.79)	0.104

Table 3 shows the effect sizes of the change scores. The Cohen's d effect sizes of the differences in the changes of both groups were in the medium range [17].

**Table 3:** Overview of effect sizes of the change scores. ^a^ EHP-5 = Endometriosis Health Profile - 5 items. ^b^ BCF = baseline carried forward. ^c^ EHP-30 = Endometriosis Health Profile - 30 items. ^d^ PDI = Pain Disability Index. ^e^ FESS = Fragebogen zur Erfassung der schmerzspezifischen Selbstwirksamkeit. ^f^ FSS = Fatigue Severity Scale. ^g^ PHQ-9 = Patient Health Questionnaire. ^h^ GAD-7 = Generalized Anxiety Disorder Scale.

Parameter	Change within Endo-App			Change within control			Comparison change, Endo-App vs. control		
	T0-T4	T0-T8	T0-T12	T0-T4	T0-T8	T0-T12	T0-T4	T0-T8	T0-T12
EHP-5^a^	0.91	0.84	0.92	0.38	0.36	0.42	0.54	0.56	0.61
EHP-5 (baseline carried forward, BCF^b^)	0.79	0.75	0.84	0.37	0.37	0.39	0.47	0.46	0.52
EHP-30^c^	0.86	0.83	0.89	0.39	0.38	0.41	0.70	0.58	0.67
EHP-30 (BCF)	0.71	0.67	0.75	0.37	0.36	0.34	0.58	0.47	0.59
PDI^d^ sum value	0.51	0.53	0.56	0.14	0.17	0.15	0.52	0.50	0.59
PDI sum value (BCF)	0.42	0.47	0.47	0.13	0.13	0.11	0.47	0.50	0.56
FESS^e^ sum value	0.43	0.49	0.52	0.08	0.05	0.18	0.53	0.64	0.47
FESS sum value (BCF)	0.35	0.44	0.47	0.07	0.03	0.09	0.45	0.61	0.55
FSS^f^ total value	0.38	0.44	0.38	0.06	0.07	0.16	0.80	0.81	0.75
FSS sum value (BCF)	0.32	0.37	0.36	0.06	0.16	0.22	0.70	0.73	0.83
PHQ-9^g^ sum value	0.44	0.42	0.56	0.04	0.02	0.04	0.62	0.61	0.86
PHQ-9 sum value (BCF)	0.36	0.36	0.45	0.02	0.02	0.02	0.55	0.54	0.74
GAD-7^h^ sum value	0.31	0.32	0.45	0.34	0.17	0.16	0.01	0.25	0.41
GAD-7 sum value (BCF)	0.26	0.31	0.45	0.35	0.17	0.14	0.02	0.24	0.45

Endometriosis Health Profile (EHP)

Regarding the EHP-30, there were significant differences between the group with the Endo-App application and the control group regarding the EHP total change score from T0 to T12 (p_U_ = 0.004). The difference between the groups was 9.15 ± 2.93 (confidence interval (CI): 3.3-15.0) in favor of the intervention group with a Cohen's d effect size of 0.67.

In the group using the Endo-App, a significant difference was found in all five EHP domains from time T0 to T12. In the control group, a significant difference was found in three of the five EHP domains from time T0 to T12. In four of the five EHP domains, there was a significant difference in the change score between the group with the Endo-App application and the control group from time T0 to T12 in favor of the Endo-App group.

There were significant differences between the Endo-App group and the control group in terms of the EHP-5 change score from time T0 to T12 (p_U_ = 0.008). The difference between the groups was 10.04 ± 3.48 (CI: 3.1-17.0) in favor of the intervention group (mean = -16.76) compared to the control group (mean = -6.72) with a Cohen's d effect size of 0.61.

The modular questionnaires “Sex” and “Work” showed a significant amelioration in the group with the Endo-App application but not in the control group. The modular segment “Doctor” did not change significantly.

Pain

There were significant differences between the group with the Endo-App application and the control group regarding the change score of the FESS sum value from time T0 to T12 (p_U_ = 0.029). The difference between the groups was 3.78 ± 1.74 (CI: 0.3-7.3) in favor of the intervention group.

There were significant differences between the group with the Endo-App application and the control group regarding the change score of the sum value of the PDI from time T0 to T12 (p_U_ = 0.007). The difference between the groups was 6.27 ± 2.35 (CI: 1.6-11.0) in favor of the intervention group.

Fatigue

There were significant differences between the group with the Endo-App application and the control group regarding the change score of the FSS sum value from time T0 to T12 (p_U_ = 0.003). The difference between the groups was 5.57 ± 1.60 (CI: 2.3-8.8) in favor of the intervention group.

Emotional well-being

There were significant differences between the group with the Endo-App application and the control group regarding the change score of the sum value of the PHQ-9 from time T0 to T12 (p_U_ < 0.001). The difference was 3.24 ± 0.80 (CI: 1.7-4.9) in favor of the intervention group.

There were no significant differences between the group with the Endo-App application and the control group regarding the change score of the sum value of the GAD-7 from time T0 to T12 (p_U_ = 0.104). The difference between the groups was 1.53 ± 0.79 (CI: 0.0-3.1) in favor of the intervention group.

Pain medication

The use of pain medication in the last 28 days was examined. For this purpose, the participants were asked on how many days they took pain medication.

There were no statistically significant differences between the group with the Endo-App application and the control group at the time points T0-T8 regarding the number of days with the use of pain medication (p_U_ = 0.572).

There were significant differences at time point T12 between the group with Endo-App application and the control group in terms of the number of analgesics/total (p_U_ = 0.015), in favor of the intervention group.

Sensitivity analyses

With conservative evaluation using the BOCF method, there were significant differences between the group with Endo-App application and the control group for EHP-5, EHP-30, FESS, PDI, PHQ-9, and FSS from time T0 to T12 in favor of the intervention group.

Adherence

At each time point, the patients were asked about their use of the app by means of a questionnaire. A total of 44 participants in the group with Endo-App application (44 of 45; 97.8%) confirmed the use of the Endo-App at T4. At T8 and T12, there were 33 and 29 people, respectively, who confirmed app use. One person stated at T4 that they could not use the app, and at T8 and T12 there were six persons each.

Over 50% of the participants reported using the Endo-App at least four days a week for at least five to 10 minutes. At T4, 72.7% of the respondents confirmed that they used the app at least four days a week, at T8 63.6%, and at T12, 58.6%. None of the respondents, and one respondent at T8 and T12, stated that they had used the app for one minute or less per day.

## Discussion

After the intervention period of 12 weeks, significant changes in the various study outcomes were shown in the group using the Endo-App. The quality of life of the Endo-App users improved significantly. After 12 weeks, changes in quality of life were not only significant but also clinically relevant. The mean change over 12 weeks in EHP-5 (mean = -16.76) exceeded the minimum clinically important difference (MCID = 4.5) by more than three times [18]. The significant and clinically relevant difference between the groups in terms of EHP remained even in a conservative sensitivity analysis with imputation, showing the robustness of the results.

Clinically relevant improvements within the intervention group were also shown for pain disability in everyday life, pain-specific self-efficacy, fatigue, and depression [19,20].

The improvements in pain disability and pain self-efficacy due to the Endo-App help explain the improvement in quality of life. It was shown that pain and the way of dealing with pain have a direct impact on quality of life [21,22]. Just like pain, fatigue as a typical side effect of endometriosis is an important influence on quality of life [23]. The overall increased prevalence of mental disorders such as depression and anxiety in people with endometriosis correlates with quality of life too [24,25]. Depressive symptoms were significantly reduced in the intervention group. Anxiety symptoms, measured with the GAD-7, tended to be reduced during the study period. From long-term studies in the literature, it can be assumed that the effects of the Endo-App continue to increase over time [26].

It should be noted that the control group also showed a significant improvement in EHP. For some other outcomes, the control group also showed a positive trend during the intervention period. On the one hand, participation in the study itself could have led to improvements through the impulse for stronger self-management. On the other hand, as shown in studies for psychological apps, a digital placebo effect may have led to improvements in the control group [27]. Despite the improvements achieved in the control group, the results in the group comparison show a clear superiority in favor of the intervention.

There were no differences between the groups regarding the number of days with pain medication at the beginning. However, after 12 weeks, the intervention group had significantly fewer days with pain medication. These results show that the improvements in the intervention group are not due to an increase in pain medication usage. Furthermore, they suggest that the use of the Endo-App can reduce pain medication usage in the long term. A long-term effect rather than a short-term effect can be expected, as patients with endometriosis often endure many years of severe pain. This can lead to complex neurological changes that exacerbate the pain [2]. It can be assumed that the improvements found in this study, e.g., in self-efficacy, quality of life, and psychological symptoms, are achieved more quickly, leading to a long-term effect on pain, the cycle of pain, and pain medication usage [26].

Overall, the bias of the study can be considered low [28]. Regarding the randomization process, the allocation sequence was random and concealed. However, the simple, unconstrained randomization resulted in unbalanced group sizes. Investigations on the success of randomization were performed. At baseline (T0), homogeneity between groups was demonstrated both demographically and in terms of baseline values of outcomes.

The study was conducted without blinding of study participants. However, allocation concealment during automated randomization ensured that everyone involved was blinded except for the study participants. All care providers, e.g., treating physicians or support staff of the manufacturer, could not see the group allocation. There was a low risk for deviations from intended interventions because of the standardized digital method of the intervention.

To exclude bias due to missing outcome data, a sensitivity evaluation using the BOCF method was conducted. There was more missing data in the intervention group compared with the control group. Both the amount of missing data and the larger number of patients lost to follow-up in the intervention group were comparable to other studies with digital health apps [29,30]. Further analyses showed that missing data did not differ significantly with respect to demographic or clinical parameters.

The methods of measuring outcomes are considered appropriate and were the same for both groups as standardized online questionnaires. Statistical analysis was performed by a third-party mathematician. The study was analyzed in accordance with the plan, that was specified before data collection.

Finally, a limitation of this pilot study is its exploratory nature. To validate the findings, a larger confirmatory randomized trial should be conducted.

## Conclusions

The care situation for endometriosis patients in Germany is insufficient. The present results show that the Endo-App could improve endometriosis care as a holistic treatment support. It was shown that the Endo-App is suitable for improving the quality of life of those affected. The Endo-App can also contribute to improving the visibility of endometriosis in the public sphere, which can have further positive effects on research and care related to the disease. As this pilot study showed the feasibility and significant results, the next step is a larger confirmatory randomized trial.

## Appendices

The list of abbreviations used in the article can be found in Table 4.

**Table 4:** Abbreviations.

Abbreviation	Full term
BCF	Baseline carried forward
BOCF	Baseline-observation-carried-forward
CI	Confidence interval
EHP	Endometriosis Health Profile
EHP-30	Endometriosis Health Profile - 30 items
EHP-5	Endometriosis Health Profile - 5 items
FESS	Fragebogen zur Erfassung der schmerzspezifischen Selbstwirksamkeit
FSS	Fatigue Severity Scale
GAD-7	Generalized Anxiety Disorder Scale
HeiQ	Health Education Impact Questionnaire
HrQoL	Health-related quality of life
ITT	Intention-to-treat analysis
MCID	Minimum clinically important difference
PDI	Pain Disability Index
PHQ-9	Patient Health Questionnaire
PSEQ	Pain Self-Efficacy Questionnaire
p_Chi_	p-value of the chi-square test
p_exF_	p-value of the Fisher's exact test
p_U_	p-value of the Mann-Whitney U test
SPSS	Statistical Package for the Social Sciences
